# Cobalt ions induce chemokine secretion in a variety of systemic cell lines

**DOI:** 10.3109/17453674.2010.537806

**Published:** 2010-11-26

**Authors:** Brian M Devitt, Joseph M Queally, Mihai Vioreanu, Joseph S Butler, David Murray, Peter P Doran, John M O'Byrne

**Affiliations:** ^1^UCD Clinical Research Centre, UCD School of Medicine and Medical Sciences, Mater Misericordiæ University Hospital; ^2^Department of Trauma and Orthopaedic Surgery, Royal College of Surgeons in Ireland, Cappagh National Orthopaedic Hospital, Dublin, Ireland

## Abstract

**Background and purpose:**

Metal ion toxicity both locally and systemically following MoM hip replacements remains a concern. Cobalt ions have been shown to induce secretion of proinflammatory chemokines locally; however, little is known about their effect systemically. We investigated the in vitro effect of cobalt ions on a variety of cell lines by measuring production of the proinflammatory chemokines IL-8 and MCP-1.

**Method:**

Renal, gastrointestinal, and respiratory epithelium and also neutrophils and monocytes were exposed to cobalt ions at 4, 12, 24, and 48 hours.

**Results:**

We found that cobalt ions enhanced the secretion of IL-8 and MCP-1 in renal epithelial cells, gastric and colon epithelium, monocytes and neutrophils, and small airway epithelial cells but not in alveolar cells. Secretion of IL-8 and MCP-1 was markedly elevated in renal epithelium, where a 16-fold and 7-fold increase occurred compared to controls. There was a 6-fold and 4-fold increase in IL-8 and MCP-1 secretion in colon epithelium and a 4-fold and 3-fold increase in gastric epithelium. Small airway epithelial cells showed a maximum increase in secretion of 8-fold (IL-8) and of 4-fold (MCP-1). The increase in chemokine secretion observed in alveolar cells was moderate and did not reach statistical significance. Monocytes and neutrophils showed a 2.5-fold and 2-fold increase in IL-8 secretion and a 6-fold and 4-fold increase in MCP-1 secretion at 48 and 24 hours, respectively.

**Interpretation:**

These data demonstrate the potent bioactivity of cobalt ions in a variety of cell types and the potential to induce a proinflammatory response.

In an attempt to reduce polyethylene-induced osteolysis and improve implant longevity, there has been renewed interest in the use of metal-on-metal (MoM) arthroplasty. The improved wear performance profiles result in substantially less wear debris and subsequent periprosthetic osteolysis (Glant et al. 1993, Shanbhag et al. 1995, Yao et al. 1995). As a result, many MoM implants have been shown to last over 2 decades, or are still functioning in patients who received the implant at a young age (Schmalzried et al. 1996). MoM bearings are now commonly used in younger patients where implant longevity is particularly important (Haynes et al. 1993). However, metal ion toxicity—both locally in the periprosthetic space and systemically—remains a concern (Horowitz et al. 1996, Haynes et al. 1997, Fritz et al. 2005, 2006). It has been well established that substantial levels of metallic products can be transferred into the host environment from implanted metallic devices (Coleman et al. 1973, Merritt and Brown 1993). Cobalt-chrome-molybdenum is the alloy used for MoM bearings, due to its wear performance profile. Cobalt and chromium ions are produced as a result of the MoM articulation (Cobb et al. 2006). In this study, we have focused on the effect of cobalt ions, as we have previously demonstrated a greater level of toxicity of these ions compared to chromium ions when in contact with primary human osteoblasts (Queally and Devitt et al. 2009).

Local effects of cobalt ions in the periprosthetic tissue include cytotoxicity, and these ions have been demonstrated to induce apoptosis, necrosis, and chemokine secretion (interleukin-8 (IL-8) and monocyte chemoattractant protein-1 (MCP-1)) and tumor necrosis factor-α secretion in both macrophages and osteoblasts (Merrit et al. 1993, Catelas et al. 2001, 2003, 2005, Huk et al. 2004, MacDonald 2004, Petit et al. 2004, Fleury et al. 2006, Keegan et al. 2007, Queally and Devitt et al. 2009). In comparison, the systemic effect of metal ions is relatively unknown. Cobalt ions can be generated in the periprosthetic space due to electrochemical corrosion of metal particles or possibly as a result of phagocytosis of CoCrMo particles by cells and exposure of these particles to a series of oxidative mechanisms designed to destroy the foreign body (Galle et al. 1992, Lundborg et al. 1992, Case 2001). The ions then bind to serum proteins (mainly albumin) and are transported systemically before being excreted in the urine (Catelas et al. 2001, Willert et al. 2005, Rasquinha et al. 2006, Grubl et al. 2007). Elevated serum levels of cobalt ions have been demonstrated, but the clinical implications of this have not been fully elucidated yet (Catelas et al. 2003, Petit et al. 2004).

The cells chosen for our experiments are representative of 3 main organ systems that can be susceptible to chronic inflammation: renal, gastrointestinal, and respiratory. Cobalt ions are excreted through the renal and gastrointestinal systems (Cobb and Schmalzreid 2006). In the distal tubule of the kidney, proximal tubule epithelial cells are in direct contact with cobalt ions during excretion. The gastrointestinal epithelial cells are avascular but are in intimate contact with the gastric submucosa and colon submucosa, which possess arterial and venus plexuses that supply and drain the vessels of the mucosa, respectively. The diffuse blood supply within this layer enables the passage of ions to the mucosa. The respiratory system was also investigated, as previous studies have shown an increased incidence of asthma and inflammatory conditions following occupational exposure to cobalt (Nemery 1990). The respiratory system can be divided into two major parts, a conducting portion and a respiratory portion. For the purpose of our experiments, two varieties of cells representative of each portion were chosen.

Neutrophils and monocytes represent the first line of defense against acute inflammatory processes. As the cobalt ions are in intimate contact with these constituents of blood during their transit through the vascular system, we also wanted to examine the effect that these ions have on monocytes and neutrophils.

The aim of our study was to investigate the in vitro effect of cobalt ions on a variety of human cell lines representing potential systemic targets of cobalt ion toxicity. The primary aim was to examine the inflammatory response elicited by cobalt ions when exposed to these cells. To achieve this, we examined IL-8 and MCP-1, two immediate-early stress response chemokines that function to attract neutrophils and monocytes respectively. These chemokines initiate the progression of an inflammatory response, which is governed to a large extent by the continued availability of infiltrating leukocytes that are recruited to the site of inflammatory injury. Previous studies have shown that cobalt ions lead to increased chemokine secretion in primary human osteoblasts with the potential to induce osteolysis by recruiting inflammatory leukocytes (Queally and Devitt et al. 2009). We hypothesized that cobalt ions have a similar pro-inflammatory effect on other systemic cell lines, thus potentially inducing an inflammatory condition or exacerbate an existing one. Specifically, we investigated the production of proinflammatory chemokines (IL-8 and MCP-1) by these cell types in response to cobalt ion exposure.

## Materials and methods

### Cell culture and metal ion exposure (Table 1)

A variety of cell lines were chosen to represent different organ systems. The cells were cultured as monolayers in their respective recommended media, in 6-well plates. All experiments were carried out once the cells had reached 80–90% confluence and at their optimal passage number. The cells were stimulated with 10 parts per million (ppm) cobalt ions (Co²^+^) (CoCl_2_; Sigma-Aldrich) in growth medium (Table 1). This concentration has been used in vitro by other authors in assessing the effect of cobalt ions on macrophages (Catelas et al. 2001, 2005, Petit et al. 2004, Huk et al. 2004, Fleury et al. 2006). Each experiment was carried out in triplicate. Supernatants were collected at 4, 12, 24, and 48 h. The medium alone without Co^2+^ was used as a negative control for each cell line.

**Table 1.** Cell cultures and culture medium used

Cell type	Medium	Passage no.
HK-2	DMEM/F12 (1:1), EGF, 10% FBS	12
AGS	Ham's F12, L-glutamine, 10% FBS	18
T-84	DMEM/F12 (1:1), L-glutamine, 10% FBS	24
SAE	Small Airway Medium with bullet kit (CC-3118, Cambrex, UK)	8
A-549	DMEM, 10% FBS	12
Neutrophils	RPMI medium 1640, 10% FBS	1
Monocytes	RPMI medium 1640, 10% FBS	1

DMEM: Dulbecco's modified Eagles medium; F12: nutrient mixture; EGF: epidermal growth factor; FBS: fetal bovine serum; RPMI: Roswell Park Memorial Institute.

### Epithelial cells of the renal proximal tubule

Human Kidney-2 (HK-2) (American Type Culture Collection (ATCC) CRL-2190) cells are an immortalized proximal tubule epithelial cell line derived from normal adult kidney, thus providing an ideal means to assess the mechanism of proximal tubule cell physiology and pathophysiology (Ryan et al. 1994).

### Epithelial cells of the gastrointestinal tract

The in vitro cells used to represent gastric epithelium were the AGS cell line (ATCC CRL-1739), which is derived from gastric adenocarcinoma (Barranco et al. 1983). In addition, T84 cells derived from colonic carcinoma were used. These cells have been found to show very similar physiological characteristics to those of normal colon epithelium, both morphologically and functionally (Dharmsathaphorn et al. 1984).

### Epithelial cells of the respiratory tract

Two kinds of cells were used, each one representative of each portion of the respiratory tract: human small airway epithelial cells (representing the conducting portion) and alveolar epithelial cells (representing the respiratory portion).

Human small airway epithelial cells (ScienCell 3230) are located at the interface between the alveoli and the conducting airways. Airway epithelial cells, which form a continuous lining of the airways, have a unique role as a protective physical and functional barrier to deleterious external agents. These cells are ideal cultures for experimental applications in asthma, inhalation toxicology, and pulmonary inflammatory responses (Crapo et al. 1983).

A549 cells (ATCC CCL-185) are human alveolar basal epithelial cells. A549 cells fall under the squamous subdivision of epithelial cells, which are associated with the diffusion of water, electrolytes, and other substances. The cell line was derived from a lung carcinoma established from an explanted lung tumor (Giard et al. 1973).

### Human neutrophils and monocytes

Circulating neutrophils and monocytes were extracted from the whole blood of healthy donors by Histopaque density centrifugation as previously described (Boyum 1968). Whereas monocytes were stimulated with cobalt ions (10 ppm) at time points up to 48 h, neutrophils were only exposed for up to 24 h due to their shorter half-life.

### Preparation and analysis of secreted protein

Enzyme-linked immunosorbent assays (ELISAs) for the detection of “free” IL-8 and MCP-1 were carried out on the supernatant samples according to the manufacturer's protocol (Promocell).

### Statistics

For studies on chemokine response, the data shown are from 1 of 3 experiments, all of which yielded similar results. For any given experiment, each data point represents the mean + SD of 6 individual cultures. Data were analyzed by one-way ANOVA using Analyze-it software. Only p-values ≤ 0.05 were considered significant.

## Results

The results of chemokine secretion by the different cell lines in response to treatment with cobalt ions are summarized in Table 2.

**Table 2.** Apprixomate fold increase in chemokine secretion relative to contrtol levels

	Length of exposure of cell cultures							
	4 hours		12 hours		24 hours		48 hours	
Cell type	IL-8	MCP-1	IL-8	MCP-1	IL-8	MCP-1	IL-8	MCP-1
Renal epithelium	2	0	14 **^a^**	5 **^a^**	16 **^a^**	7 **^a^**	15 **^a^**	7 **^a^**
Gastric epithelium	2	1.2	2.5	2.5 **^a^**	3.7 **^a^**	3 **^a^**	4 **^a^**	3 **^a^**
Colonic epithelium	1.8	0	1.8	0	4 **^a^**	3.3 **^a^**	6 **^a^**	4 **^a^**
SAE	2.5	1.3	3	1.5	5 **^a^**	4 **^a^**	8 **^a^**	4 **^a^**
Alveolar epithelium	1.5	0	2	0	2	0	2	2
Neutrophils	0	2.5 **^a^**	2 **^a^**	4 **^a^**	2 **^a^**	4 **^a^**	N/A	N/A
Monocytes	1.5	1.6	2 **^a^**	3	2.5 **^a^**	4	2.5 **^a^**	6

**^a^** p < 0.05 SAE: small airway epithelium; N/A: no available timpe point.

### Cobalt ions stimulated IL-8 and MCP-1 secretion in renal epithelial cells

**Figure 1.**
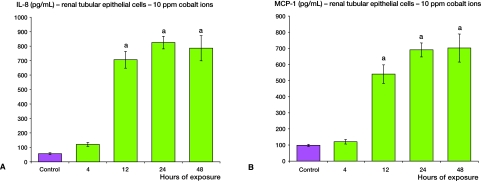
Cobalt ions (10ppm) induce enhanced secretion of IL-8 and MCP-1 chemokines in human renal tubular epithelial cells (HK-2) at 12, 24 and 48 h. Renal tubule epithelial cells were treated with 10 ppm cobalt ions for 4, 12, 24 and 48h along with a negative control. A: There is a significant increase in the secretion of IL-8 protein post exposure at 12, 24 and 48 h relative to the control sample (a = p < 0.001). B: There is also a significant increase in the secretion of MCP-1 protein at 12, 24, and 48h compared to negative control (a = p < 0.001).

Following 12, 24, and 48 h of treatment with cobalt ions (at 10 ppm), renal tubular epithelial cells showed enhanced secretion of both IL-8 and MCP-1 relative to the control sample (p < 0.001) (Figure 1). A 16-fold maximal increase was observed for IL-8 secretion, whereas the maximal increase for MCP-1 secretion was 7-fold.

### Cobalt ions stimulated secretion of IL-8 and MCP-1 in gastrointestinal epithelial cells

**Figure 2.**
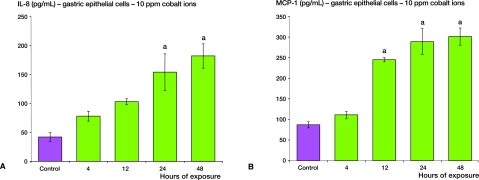
Cobalt ions (10ppm) induce enhanced secretion of IL-8 and MCP-1 chemokines in human gastric epithelial cells (AGS). Gastric epithelial cells were treated with 10 ppm cobalt ions for 4, 12, 24 and 48h along with a negative control. A: There is a significant increase in the secretion of IL-8 protein post exposure at 24 and 48h relative to the control sample (a = p < 0.001). B: There is also a significant increase in the secretion of MCP-1 protein at 12, 24, and 48h compared to negative control (a = p < 0.001).

**Figure 3.**
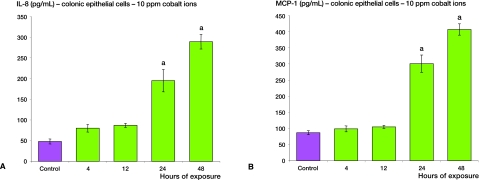
Cobalt ions (10ppm) induce enhanced secretion of IL-8 and MCP-1 chemokines in human colonic epithelial cells (T-84). Colonic epithelial cells were treated with 10 ppm cobalt ions for 4, 12, 24 and 48h along with a negative control. A: There is a significant increase in the secretion of IL-8 protein post exposure at 24 and 48h relative to the control sample (a = p < 0.001). B: There is also a significant increase in the secretion of MCP-1 protein at 24 and 48h compared to negative control (a = p < 0.001).

Following treatment with cobalt ions (10 ppm), gastric epithelial cells showed enhanced secretion of both IL-8 and MCP-1 relative to the control sample (p < 0.001) (Figure 2). A 4-fold maximal increase was observed for IL-8 secretion and a 3-fold maximal increase was observed for MCP-1 secretion. A similar increase in chemokine secretion was observed in colon epithelial cells. Following 24 and 48 h of treatment with cobalt ions (10 ppm), colon epithelial cells showed enhanced secretion of both IL-8 and MCP-1 relative to the control sample (p < 0.001) (Figure 3). A 6-fold maximal increase was observed for IL-8, whereas the maximal increase in MCP-1 secretion was 4-fold.

### Cobalt ions stimulated secretion of IL-8 and MCP-1 in small airway epithelial cells but not in alveolar epithelial cells

**Figure 4.**
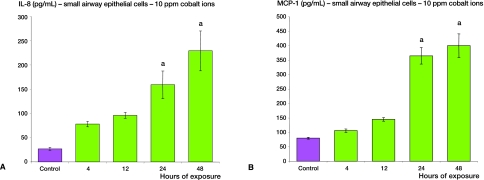
Cobalt ions (10ppm) induce enhanced secretion of IL-8 and MCP-1 chemokines in human small airway epithelial cells. Human small airway epithelial cells were treated with 10 ppm cobalt ions for 4, 12, 24 and 48h along with a negative control. A: There is a significant increase in the secretion of IL-8 protein post exposure at 24 and 48h relative to the control sample (a = p < 0.001). B: There is also a significant increase in the secretion of MCP-1 protein at 12, 24 and 48h compared to negative control (a = p < 0.001).

Following 24 and 48 h of treatment with cobalt ions (10 ppm), maximal increases of 8- and 4-fold were observed in the secretion of IL-8 and MCP-1, respectively, by small airway epithelial cells (p < 0.001) (Figure 4). An increase in chemokine secretion was observed in alveolar epithelial cells but this increase was not statistically significant (p = 0.06) (Figure 5, supplementary data).

### Cobalt ions stimulated secretion of IL-8 and MCP-1 in neutrophils and monocytes

Following treatment with cobalt ions (10 ppm), neutrophils showed enhanced secretion of both IL-8 and MCP-1 relative to the control sample (p < 0.001) (Figure 6, supplementary data). A 2-fold maximal increase was observed for IL-8 whereas the maximal increase in MCP-1 secretion was 4-fold. A similar increase in chemokine secretion was observed in monocytes. Following treatment with cobalt ions (10 ppm), monocytes showed enhanced secretion of both IL-8 and MCP-1 relative to the control sample (p < 0.001) (Figure 7, supplementary data). A 2.5-fold maximal increase was observed for IL-8 whereas the maximal increase in MCP-1 secretion was 6-fold.

## Discussion

Metal-on-metal articulations consist of a cobalt-chromium-molybdenum alloy and are known to release wear particles and corrosion products into the periprosthetic tissue; they are also transported from the joint and distributed systemically (Coleman et al. 1973, Urban et al. 2000, Jacobs et al. 2004). There is a measurable increase in cobalt ion levels in the serum, erythrocytes, and urine of patients with MoM bearings (Coleman et al. 1973, Jacobs et al. 1998, Savarion et al. 2002, Brodner et al. 2003, MacDonald et al. 2003, Rasquinha et al. 2006).

The potential effects of elevated levels of metal ions are poorly defined. A number of adverse biological reactions have been linked to the dissemination of metal ions, including soft tissue toxicity, hypersensitivity reactions, bone loss, and risk of carcinogenesis (Gillespie et al. 1996, Shimmin et al. 2005, Willert et al. 2005, Keegan et al. 2007, Lidgren 2008). In vitro studies have clearly demonstrated that cobalt particles can be highly cytotoxic when produced in high enough concentrations (Merritt and Brown 1993). There is also much concern about the effect of long-term exposure and the potential for chromosomal aberrations in the patient, as well as the risk of passing chromosomal abnormalities to the next generation (Case et al. 1996, Doherty et al. 2001, Ladon et al. 2004, Bordner et al. 2004a, Papageorgiou et al. 2007, Ziaee et al. 2007).

The inflammatory response to a foreign material is of particular relevance. This is the area on which we have focused our research. The response to a foreign material involves a cascade of events. The typical inflammatory response is marked by the accumulation of polymorphonuclear leukocytes at the implant site. We have previously demonstrated that osteoblasts play an integral role in the initiation of this process by secreting proinflammatory chemotactic cytokines, which are sufficient to induce the migration of monocytes and neutrophils (Queally and Devitt et al. 2009). However, metal ions are not restricted to the periprosthetic space and they have direct access to other tissues as result of their movement through the blood. In this study, we have investigated the specific chemokine responses in a variety of human cell lines that represent potential systemic targets of cobalt ion toxicity, following exposure to cobalt ions (Firriolo et al. 1999, Urban et al. 2000, IARC Monographs) Chemokines are the major mediators of chemotactic signaling in the recruitment and activation of inflammatory cells (Miller et al. 1992, Taub et al. 1994). IL-8 and MCP-1 are immediate-early stress response chemokines that function to attract neutrophils and monocytes, respectively.

Unlike most organic chemicals, metals cannot be eliminated from tissues by metabolic degradation. Thus, they can only be eliminated from tissues by renal or gastrointestinal excretion (Cobb and Schmalzreid 2006). It is widely regarded, although without evidence-based research, that MoM bearing surfaces are contraindicated in patients with chronic renal failure. In such cases, there is a potential for elevated cobalt levels within the circulation as a result of reduced excretion (Brodner et al. 2000). However, of equal concern is the effect that cobalt ions have on renal tissue and whether this effect contributes to renal failure in itself. In light of this, we explored the effect of exposure of proximal tubule epithelial cells to cobalt ions. In this study, we have demonstrated for the first time that cobalt ions enhance protein secretion of IL-8 and MCP-1 in renal proximal tubule epithelial cells. The enhanced secretion was substantial, representing a 16-fold and 7-fold increase, respectively, compared to control levels.

Proinflammatory mediators recruit neutrophils and monocytes to the site of insult, which drive the inflammatory response. In the setting of chronic renal failure, the added insult of a further inflammatory stimulus may be sufficient to tip the balance into a vicious circle of reduced excretion and increased circulating levels of cobalt ions. The effect may also be compounded by the fact that neutrophils and monocytes, having been recruited, also give rise to enhanced IL-8 and MCP-1 secretion following exposure to cobalt ions (10 ppm). Whether this is sufficient to induce full renal failure remains to be seen, but it is certainly an area of concern and warrants further research.

Urban et al. have previously demonstrated the dissemination of wear particles to the liver, spleen, and abdominal lymph nodes of patients with a hip or knee replacement following retrieval studies (Urban et al. 2000). The gastrointestinal tract is intimately related to these three routes of dissemination, via the portal venous system and lymphatic drainage (Strandring 2008). In the present study, elevated secretion of these proinflammatory chemokines was seen in gastric and colon epithelium. Gastric epithelium showed a 4-fold and 3-fold maximal enhanced secretion of IL-8 and MCP-1, respectively, while colon epithelium showed a maximal 6-fold increase in IL-8 secretion and a 4-fold increase in MCP-1 secretion. It is difficult to assess the importance of this enhanced secretion from gastrointestinal epithelium. A link may perhaps be drawn between this and another chronic relapsing inflammatory condition that can occur in the gastrointestinal tract, inflammatory bowel disease. This disease is characterized by continuous infiltration of affected tissue by inflammatory cells from the circulation. This influx results in further tissue-destructive inflammatory processes. The recruitment and activation of immunocytes is mediated by chemokines, including IL-8 and MCP-1 (Uguccioni et al. 1999, McCormack et al. 2001). Chemokine secretion is low or non-existent in resting cells but rapidly becomes upregulated during inflammation (Luster 1998). The potential therefore exists for quiescent inflammatory bowel disease to become reactivated as a result of an inflammatory insult, thus perpetuating the disease process (McCormack et al. 2001). Is it then possible that exposure to high levels of cobalt ions in an individual with inflammatory bowel disease may precipitate a relapse of the inflammatory condition. This is simply a theory, and to date there have been no case reports to corroborate this. It is certainly something to consider in a young patient with inflammatory bowel disease requiring a total hip replacement, perhaps as a result of steroid-induced avascular necrosis of the hip (Klingenstein et al. 2005).

Frequent contact with inhaled cobalt from occupational exposure has been shown to cause an increased incidence of asthma and chronic inflammatory pulmonary conditions, either from the particles themselves or from solubilized cobalt ions (Nemery 1990, IARC Monographs). The toxic responses of the respiratory system in metal workers are largely related to inhalation exposure, and are therefore difficult to extrapolate to the vascular route. The respiratory system can be differentiated into two major parts: a conducting portion, consisting of structures that deliver air to the lungs, and a respiratory portion, consisting of structures within the lungs where oxygen is exchanged for carbon dioxide in the blood. The respiratory system has lining epithelia, supporting structures, glands, and other features that are characteristic of each part. The blood flow to the intraparenchymal airways is predominately from the bronchial circulation (Bernard et al. 1996). As a result, cobalt ions bound to proteins have the potential to come in contact with respiratory epithelium through systemic transmission. Our experiments demonstrate that exposure of small airway epithelial cells to cobalt ions can give rise to an 8-fold increase in IL-8 secretion and a 4-fold increase in MCP-1 secretion. Regarding human alveolar epithelial cells, roughly a 2-fold (maximal) increase in both IL-8 and MCP-1 secretion was observed, but this did not reach statistical significance. These results suggest that the conduction portion of the respiratory system induces an inflammatory response following exposure to cobalt ions while the respiratory portion remains relatively quiescent. Once again, the significance of these data in the clinical setting is unknown and to date there have been no case reports documenting any deterioration in chronic pulmonary conditions following MoM hip replacement. Further work is required to determine the long-term implications of elevated cobalt ion levels in patients with pulmonary disease.

Finally, we explored the effect of exposure of monocytes and neutrophils found within the bloodstream to cobalt ions. We found that monocytes have a rapid response to cobalt ions, inducing the secretion of IL-8 and MCP-1 at 4, 12, 24, and 48 h. Likewise, neutrophils showed a significant increase in secretion of these cytokines. These cells are found both in the circulation and deposited in tissues. They appear to have dual roles as responders to inflammation and recruiters of further leukocytes once stimulated by cobalt ions, thereby perpetuating the inflammatory cascade.

Metallic debris may exist as particles, in ionic form, or as inorganic salts. In this study, we used cobalt ions at a concentration of 10 ppm. The exact concentration of these ions in the periprosthetic space and systemically remains unclear, as there has been variation in study design, in the sources of samples (serum, whole blood, erythrocytes, and urine), and in the method of laboratory analysis (Catelas et al. 2005). This concentration has been used in vitro by other authors in assessing the effect of cobalt ions on macrophages (Catelas et al. 2001, 2005, Huk et al. 2004, Petit et al. 2004, Fleury et al. 2006). Serum blood levels are less than 10 ppm; however, these higher levels may be seen in patients with “runaway” wear (see below) or in the setting of renal failure (Hur et al. 2008, Lee et al. 2008). There is recent evidence to suggest that cobalt levels are influenced by factors such as the type, design, and positioning of the implant (Savarino et al. 2002, Brodner et al. 2003, Back et al. 2005a). The position of the implant, which is influenced by the skill of the surgeon, plays a major role in the degree of metal ion release. Brodner reported a 10- to 53-fold increase in cobalt levels in patients with an acetabular cup inclination of between 58° and 63° (Brodner et al. 2004b). The classical “run-in” period is well recognized with MoM prostheses, as peak serum levels of cobalt show a 10-fold increase at six months relative to the preoperative levels (Back et al. 2005b, Daniel et al. 2007). These peaks are followed by a gradual decline over the next 15 months as the bearing surface enters “steady-state” wear. Bowsher et al. (2009) have also described the concept of “runaway” wear, which is a puzzling phenomenon that can result in a 2- to 19-fold increase in wear in prostheses compared to identical bearings (Bowsher et al. 2009).

In summary, the data presented here demonstrate the potent bioactivity of cobalt ions in a variety of systemic cell lines. This process is most likely representative of an overall toxic effect of high-dose exposure to cobalt ions. The significance of these findings has particular relevance in the context of patients undergoing hip replacement surgery with pre-existing renal failure, where there is a greater degree of exposure to high levels of cobalt ions due to reduced excretion. It may also be important in cases where the hip implant cup inclination is placed in excessive abduction or where “runaway” wear is experienced. In addition, due consideration should be given to the choice of bearing surface in patients with inflammatory bowel disease due to the possibility of triggering a relapse.

## Supplementary data

Figures 5–7 are available at our website (www.actaorthop.org), identification number 3791.
