# Supplementary material for: Cobalt ions induce chemokine secretion in a variety of systemic cell lines
Source: Acta Orthop. 2010 Nov 26;81(6):756–64. doi: 10.3109/17453674.2010.537806 (PMC3216089; doi:10.3109/17453674.2010.537806)
Supplement: Supplementary file 1 [file ORT-1745-3674-81-756-s3791.pdf]

## Supplementary article data

# Cobalt ions induce chemokine secretion in a variety of systemic cell lines

Brian M Devitt[1], Joseph M Queally[1], Mihai Vioreanu[1], Joseph S Butler[1], David Murray[1], Peter P Doran[1], and John M O'Byrne[2]

[1]UCD Clinical Research Centre, UCD School of Medicine and Medical Sciences, Mater Misericordiæ University Hospital; [2]Department of Trauma and Orthopaedic Surgery, Royal College of Surgeons in Ireland, Cappagh National Orthopaedic Hospital, Dublin, Ireland

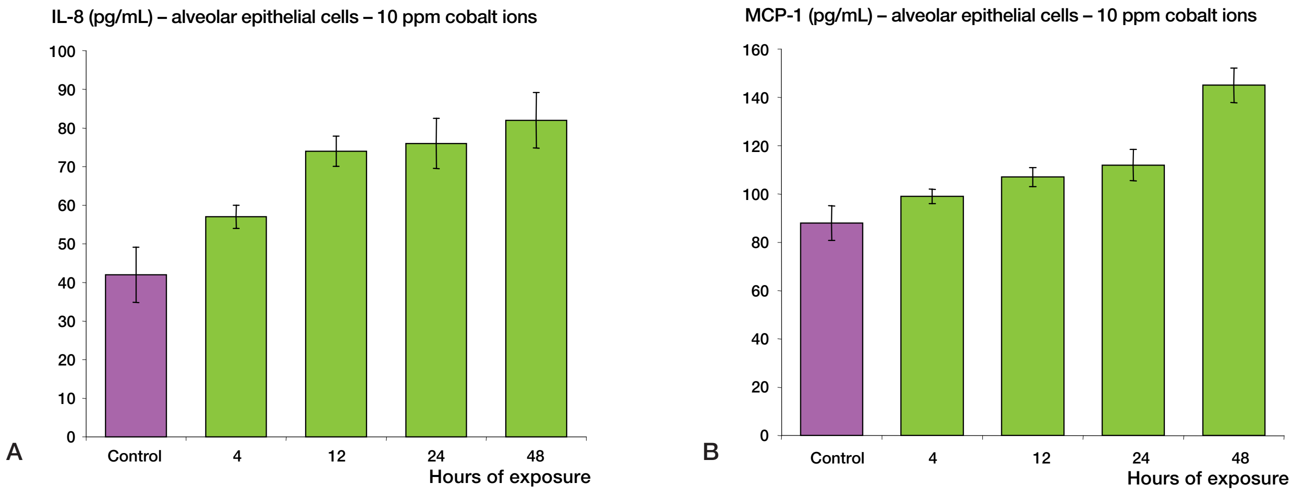


Figure 5. Cobalt ions (10ppm) have no effect on the secretion of IL-8 and MCP-1 chemokines in human alveolar cells. Alveolar epithelial cells were treated with 10 ppm cobalt ions for 4, 12, 24 and 48h along with a negative control. A: No significant was observed increase in the secretion of IL-8 protein post exposure at 12, 24 and 48h relative to the control sample ( $p > 0.05$). B: No significant increase was observed in the secretion of MCP-1 protein at 12, 24 and 48h compared to negative control ($p > 0.05$).

*Open Access - This article is distributed under the terms of the Creative Commons Attribution Noncommercial License which permits any noncommercial use, distribution, and reproduction in any medium, provided the source is credited.*
*DOI 10.3109/17453674.2010.537806*

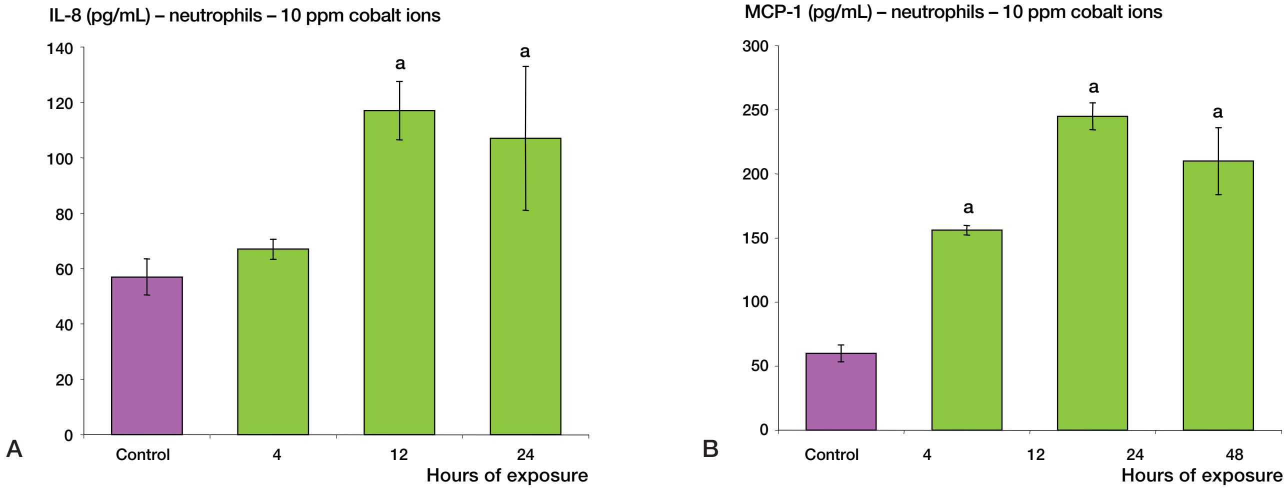


Figure 6. Cobalt ions (10ppm) induce enhanced secretion of IL-8 and MCP-1 chemokines in human neutrophils. Neutrophils were treated with 10 ppm cobalt ions for 4, 12 and 24h along with a negative control. A: There is a significant increase in the secretion of IL-8 protein post exposure at 12 and 24h relative to the control sample (a = $p < 0.001$). B: There is also a significant increase in the secretion of MCP-1 protein at 12, and 24h compared to negative control (a = $p < 0.001$).

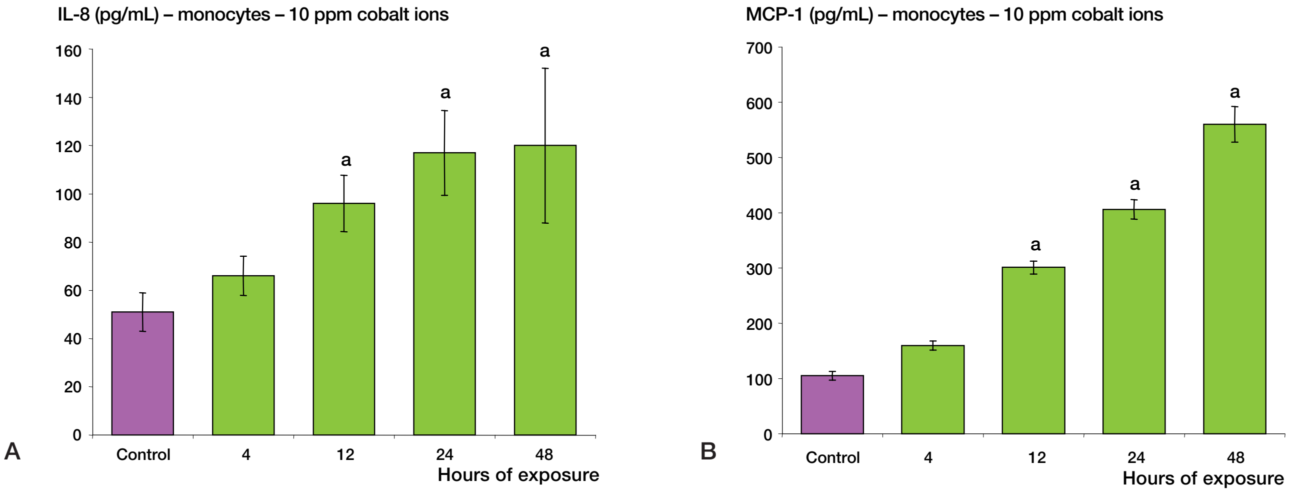


Figure 7. Cobalt ions (10ppm) induce enhanced secretion of IL-8 and MCP-1 chemokines in human monocytes. Neutrophils were treated with 10 ppm cobalt ions for 4, 12, 24 and 48h along with a negative control. A: There is a significant increase in the secretion of IL-8 protein post exposure at 12, 24 and 48h relative to the control sample (a = $p < 0.001$). B: There is also a significant increase in the secretion of MCP-1 protein at 12, 24 and 48h compared to negative control (a = $p < 0.001$).
